# Competences in the training of nurses to assist the airway of adult patients in urgency and emergency situations*

**DOI:** 10.1590/1518-8345.3380.3434

**Published:** 2021-07-02

**Authors:** Fernanda Berchelli Girão Miranda, Gerson Alves, Alessandra Mazzo

**Affiliations:** 1 Universidade Federal de São Carlos, Departamento de Enfermagem, São Carlos, SP, Brazil.; 2 Scholarship holder at the Coordenação de Aperfeiçoamento de Pessoal de Nível Superior (CAPES), Brazil.; 3 Universidade de São Paulo, Curso de Medicina de Bauru, Bauru, SP, Brazil.

**Keywords:** Nurses, Clinical Competence, Airway Management, Nursing Education, Nursing Assessment, Emergency Nursing, Enfermeiros, Competência Clínica, Manuseio das Vias Aéreas, Educação em Enfermagem, Avaliação em Enfermagem, Enfermagem em Emergência, Enfermeros, Competencia Clinica, Manejo de la Via Aérea, Educación en Enfermería, Evaluación en Enfermería, Enfermería de Urgencia

## Abstract

**Objective:**

construction and validation in appearance and content of the competence frameworks and of the Entrustable Professional Activities to develop skills in the training of nurses to assist the airway of adult patients in urgency and emergency situations.

**Method:**

a descriptive and methodological study developed in four phases: in the first, a workshop was held, composed of experts, for the construction of the competence frameworks; in the second, the material was validated using the Snowball Technique and the Delphi Technique, in the third, content analysis and calculation of the Content Validation Index were conducted; and in the fourth phase, the Entrustable Professional Activities were built, validated in simulated workshops.

**Results:**

the competence frameworks were built and validated, with a resulting CVI≥0.85 in all the items. The Entrustable Professional Activities were validated by experts regarding their applicability; of these, 44% stated they were applicable in simulated environments, 100% that they were useful content and with appropriate language, 22% suggested the insertion of new items to assess competence, 11% reported the difficulty of assessing competence individually in the clinical settings, and 11% of the experts referred to the need for prior training of the teacher/facilitator to use it.

**Conclusion:**

the study resulted in the construction of competence frameworks and six Entrustable Professional Activities relating them to the domains of essential competences in the training of nurses to assist the airway of adult patients in urgency and emergency situations. The participation of experts in the construction and validation of this material was essential to guarantee the theoretical and practical relevance of the result.

## Abstract (pt)

**Objetivo:**

construção e validação em aparência e conteúdo dos marcos de competências e das *Entrustable Professional Activities* para desenvolver as competências na formação do enfermeiro para a assistência à via aérea de pacientes adultos em urgência e emergência.

**Método:**

estudo descritivo, metodológico desenvolvido em quatro fases: na primeira realizou-se uma oficina, composta por *experts*, para a construção dos marcos de competências; na segunda houve a validação dos material através da *Snowball Technique* e Técnica Delphi, na terceira ocorreu a análise de conteúdo e cálculo do Índice de Validação de Conteúdo; e na quarta fase foram construídas as *Entrustable Professional Activities*, validadas em oficinas simuladas.

**Resultados:**

construídos e validados os marcos de competências resultando em todos os itens IVC≥0,85. As *Entrustable Professional Activities* foram validadas por *experts* quanto à sua aplicabilidade, destes 44% referiram serem aplicáveis em ambientes simulados, 100% refeririam ser um conteúdo útil e com linguagem adequada, 22% sugeriram a inserção de novos itens para avaliar a competência, 11% relataram a dificuldade de avaliar a competência individualmente nos cenários clínicos, 11% *experts* referiram a necessidade de capacitação prévia do docente/facilitador para a utilização.

**Conclusão:**

o estudo resultou na construção dos marcos de competências e seis *Entrustable Professional Activities* relacionando-os com os domínios de competências essenciais na formação do enfermeiro para a assistência à via aérea de pacientes adultos em urgência e emergência. A participação dos *experts* na construção e na validação desse material foi fundamental para garantir a relevância teórica e prática do resultado.

## Abstract (es)

**Objetivo:**

construcción y validación de la apariencia y el contenido de los marcos de competencias y de las *Entrustable Professional Activities* para desarrollar las competencias en la formación de enfermeros para asistir la vía aérea de pacientes adultos en urgencia y emergencia.

**Método:**

estudio descriptivo, metodológico desarrollado en cuatro fases: en la primera se realizó un taller, integrado por *experts*, para la construcción de marcos de competencias; en el segundo se realizó la validación de los materiales a través de la *Snowball Technique* y la Técnica Delphi, en el tercero se realizó el análisis de contenido y cálculo del Índice de Validación de Contenido; y en la cuarta fase se construyeron *Entrustable Professional Activities*, validadas en talleres simulados.

**Resultados:**

se construyeron y validaron marcos de competencias, resultando en todos los ítems IVC≥0,85. Las *Entrustable Professional Activities* fueron validadas por *experts* en cuanto a su aplicabilidad, de estos el 44% dijo que eran aplicables en entornos simulados, el 100% dijo que eran contenidos útiles y con lenguaje adecuado, el 22% sugirió la inserción de nuevos ítems para evaluar competencia, el 11% reportó dificultad para evaluar la competencia individualmente en entornos clínicos, el 11% de los *experts* reportó la necesidad de capacitación previa del docente/facilitador para utilizarla.

**Conclusión:**

el estudio resultó en la construcción de marcos de competencias y seis *Entrustable Professional Activities* relacionándolos con los dominios de competencias esenciales en la formación de enfermeros para asistir la vía aérea de pacientes adultos en urgencia y emergencia. La participación de *experts* en la construcción y validación de este material fue fundamental para asegurar la relevancia teórica y práctica del resultado.

## Introduction

Urgency and emergency (UE) situations can occur at any level of care in the health services, and the nurses, as team leaders, must be adequately prepared to deal with them in an effective, competent and systematic manner. Among the most common, those involving patients with airway involvement require a confident and immediate professional performance, since the risk of clinical instability and evolution to cardiopulmonary arrest is imminent^(^ 1 ^)^.

The faults caused during care, often due to inadequate technical or non-technical skills, and erroneous judgment in airway management, can result in high rates of death or irreversible brain damage to the patient, in addition to high costs and prolonged hospitalization periods in health institutions^(^ 2 ^-^ 3 ^)^.

Airway permeability can be effectively ensured by using relatively simple interventions, such as checking the patient's verbal response, maneuvers involving the head and jaw, thoracic inspection, and visual assessment of possible obstructions such as foreign bodies, vomiting, secretions, facial, mandibular or laryngeal fractures^(^ 1 ^,^ 4 ^)^. Nurses are among the professionals responsible for carrying out these interventions; however, they often do not receive training for airway assessment and intervention. Many *curricula* do not address the development of these skills, which hinders the practical insertion of the competence of these professionals in this area^(^ 5 ^)^. In addition, the current professional training model adopted by most Higher Education Institutions (HEIs) has resulted in an evident gap between the profile of the professionals who are entering the job market in view of the real health needs of the users^(^ 6 ^-^ 7 ^)^. Some authors point out that most novice nurses feel unable to assess signs and symptoms resulting from airway obstruction and to perform a ventilatory assessment of the patient, failing to develop fast and safe interventions^(^ 8 ^-^ 9 ^)^.

To change this situation, training and continuing education of the professionals increasingly need to develop and improve the skills necessary to minimize the number of incidents. Technical and non-technical skills must be developed to the level of excellence, without, however, harming or jeopardizing the health status of the patients^(^ 10 ^)^.

In the international literature, among the various benchmarks for the development and assessment of the skills of the health professionals in the emergency care line, the Milestones and the Entrustable Professional Activities (EPAs) stands out^(^ 11 ^-^ 13 ^)^. Competence frameworks are descriptions of the knowledge, skills and attitudes for each of the competences expected during student training. They describe in a narrative manner the competences that are developed over a period of time and must be demonstrated repeatedly throughout the student's training in clinical environments of different complexity levels^(^ 14 ^-^ 16 ^)^.

The EPAs are characterized as an evolution of the educational concept based on competences, in which the concept of competences of an apprentice is applied in specific contexts in the workplace^(^ 17 ^)^. They constitute the job description and are independent of people, operationally define a profession, establishing a list of specific tasks that must occur in a period of time which can be planned. For apprentices to become competent professionals, they must acquire skills that include knowledge, skills and attitudes^(^ 18 ^)^.

On this theme, this study aimed to build and validate the appearance and content of the competence frameworks and EPAs to develop skills in the training of nurses to assist the airway of adult patients in urgency and emergency situations.

## Method

A descriptive and methodological study of construction and validation of the competence frameworks and the EPAs to be developed in the training of nurses, for the assistance of adult patients to approach the airways in urgency and emergency situations.

In the first phase of the study a workshop was held, made up of experts, for the construction of the competence frameworks. The experts were selected using the snowball technique^(^ 19 ^)^, in which the researchers asked a teacher (key-informant) of a Public University of the inland of the state of São Paulo to indicate the name and email address of three professionals who would meet the inclusion criteria of the study. Through this data, the invitations were sent by e-mail clarifying the research objective for each of them, together with the request for new indications of possible participants, with a total of 17 professionals being contacted.

The selection of the experts took place based on Fehring's adapted referential^(^ 20 ^)^; the inclusion criteria established for this phase constitute health professionals with at least one year of experience in assistance and/or teaching with adult patients in urgency and emergency situations, having a certificate of clinical practice (specialization), a master's degree or doctoral thesis in the area of interest of the study, clinical practice of at least one year in the area of interest of the study, publication of relevant research for the area of interest or publication of an article on the topic (Nursing and/or urgency and emergency) in a reference journal (classified in strata indicative of quality by national and international Bibliometric Indicators). To be considered expert, the participant presented at least one of the items mentioned above.

During the workshop, the researchers trained the experts by means of a dialogued expository presentation, provided readings and discussions of international referentials on the theme, using the following guiding question: "What competences must be developed in training of nurses, for the assistance of adult patients to approach the airways in urgency and emergency situations?" as a trigger among the experts. Subsequently, it was requested to build competence frameworks for the training of nurses in the care of the airways for adult patients in urgency and emergency situations.

In the second phase of the study, the validation of the constructed competence frameworks was carried out. In this phase, new experts were selected through the "snowball technique"^(^ 19 ^)^, in which, again, the same key-informant indicated the name and email address of three professionals who met the inclusion criteria of the study and, in each invitation made to a new participant, this request was reproduced. For the inclusion of the experts, Fehring's adapted criteria^(^ 20 ^)^ were considered; thus, as in the previous phase, with the difference that all guests are necessarily nurses. In this phase, 76 professionals received an invitation to participate in the survey via electronic mail with an access *link* through which, when clicking, the professional was directed to the electronic form, made available by Google Docs Off line^®^, with immediate opening of the Free and Informed Consent Form (FICF) to be filled in, being a mandatory condition for opening the following pages, which presented the biographical and professional characterization form, the instruction manual for editing, and the competence frameworks to be validated in appearance and content.

The participating experts were asked to return the data collection instruments within a maximum period of 30 days. 15 professionals responded to the validation of the constructed material. To obtain consensus on the answers, the Delphi technique was used^(^ 21 ^)^.

In the third phase of the study, content analysis^(^ 19 ^)^ was performed with the categorization, classification and quantification of data for interpretation of the results, through the creation of units of significance and contextual units. Also in this phase, the Content Validation Index (CVI) was calculated to assess the judges' agreement regarding the representativeness of each item in the charts, with a minimum index of 0.80 for each item in the chart being considered acceptable^(^ 22 ^)^.

Thus, a new version of the competence frameworks was issued and, subsequently, a second round of opinions was requested from the experts. The 15 experts received a new email with the reformulated competence frameworks, with a maximum term of 30 days. 13 experts participated in this phase, finalizing the agreement of the material presented.

In the fourth phase of this study, considering the competence frameworks constructed and validated in the previous phase and in other studies^(^ 16 ^,^ 23 ^-^ 24 ^)^, six EPAs related to Nursing care in the airway in urgency and emergency situations were built. The validation regarding content, appearance and applicability was carried out after the invitation to the teachers and nurses from the educational institution for voluntary participation as experts in a workshop to present the content and objectives of the research and in the simulated workshops of the Advanced Life Support (ALS) Course for Adults offered free of charge by the unit to students of the first year of the Master's Degree in Medical-Surgical Nursing, at the Unit's Clinical Simulation Center, in the period of July 2017. The criteria established for the inclusion of experts also followed Fehring's adapted criteria^(^ 20 ^)^.

In all phases of the study, the participants signed the FICF and answered a biographical and professional characterization form. The development of the study took place from March to August 2017, after ethical authorization under Opinion No. 55082716.5.0000.5393.

## Results

As a result of the stages described above, the competence frameworks and EPAs to be developed during the training process of nurses, in assisting the airways of patients in urgent and emergency situations, were built and validated.

Below, Table 1 shows the characterization of the experts who contributed in all phases of the material construction and validation process.

**Table 1:** Characterization of the experts participating in each validation phase of the competence frameworks and the Entrustable Professional Activities (EPA). Ribeirão Preto, SP, Brazil, 2017

Variables		First stage experts f (%)	Second stage experts f (%)	Third stage experts f (%)	Fourth stage experts f (%)
	Participants	7 (100%)	15 (100%)	13 (100%)	9 (100%)
Gender					
	Male	2 (28,6%)	6 (40,0%)	5 (38,4%)	4 (44%)
	Female	5 (71,4%)	9 (60,0%)	8 (53,3%)	5 (55%)
Professional qualification					
	Nurse	6 (85,7%)	15 (100%)	13 (100%)	9 (100%)
	Physician	1 (14,3%)	0	0	0
Postgraduate studies*					
	Specialization	4 (57%)	10 (66,7%)	10 (76,9%)	7 (78%)
	Master's	5 (71,4%)	15 (100%)	13 (100%)	5 (55%)
	PhD	3 (42,8%)	7 (46,7%)	7 (53,8%)	8 (89%)
	Post-Doctorate	1 (14,3%)	1 (6,7%)	1 (7,69%)	0
Current professional practice area					
	Assistance	1 (14.3%)	4 (26.7%)	4 (30.7%)	7 (78%)
	Teaching	5 (71.4%)	6 (40.0%)	4 (30.7%)	7 (78%)
	Assistance and teaching	1 (14.3%)	5 (33.3%)	5 (38.4%)	6 (67%)
	Publications of articles on the theme in journals	6 (85.7%)	6 (40,0%)	6 (46,1%)	6 (67%)

* The experts reported more than one academic degree

In the first phase of the study, in which the construction of the competence frameworks took place, the researchers did not limit the participation of the experts to nurses only, aiming at the collaboration of other health professionals working in undergraduate courses in many HEIs in the country that could meet the proposed requirements. In this context, for this phase, obeying the proposed criteria, there was the participation of only one medical professional who worked as a teacher at a private university in Nursing education. After defining the competence frameworks, the validation process was intended only for expert nurses.

In the following phases, the experts contributed to the validation using the Delphi technique^(^ 21 ^)^. With the content analysis of the suggestions received, the judges' agreement was verified regarding the representativeness of the items in relation to the content of the tables. In the third phase, some items of the first analysis presented a CVI below 0.80; thus, the comments and suggestions of the experts were considered for the possibility of adjustments, with return to the participants, resulting in a final analysis on all items with a CVI≥0.85.

The EPAs were validated in clinical scenarios simulated by nine experts regarding their applicability, four (44%) experts reported that the content of the EPAs is applicable in simulated environments; however, 1 (11%) expert detected the content to assess the competence as very extensive and 2 (22%) experts suggested the insertion of a column with the possibility to mark yes or not in relation to the development of the expected competence, all the experts (100%) stated it was useful content and with appropriate language, 2 (22%) suggested the insertion of new items to assess the competence and 1 (11%) reported the difficulty of assessing competence individually in clinical settings, one (11%) expert referred to the need for prior training of the teacher/facilitator to use the content of the EPAs.

Figures 1 and 2 below show the final association between the competence frameworks and the domains of essential competences for the development of the EPAs, in relation to the clinical situations of urgency addressed and the descriptions of the expected behavior of each student, in which the reliability of their performance is discussed.

**Figure 1:** Association between competence frameworks to develop competences in the training of nurses for the care of adult patients in urgency and emergency care, essential competences and Entrustable Professional Activities (EPA 1, EPA 2 and EPA 3). Ribeirão Preto, SP, Brazil, 2017

Competence framework	Essential competences (Knowledge, Skill and Attitude)	Entrustable Professional Activities (EPAs)
Knows the anatomy and physiology of the upper and lower airways; performs targeted anamnesis and physical examination, and associates anamnesis and physical examination with possible Nursing diagnoses.	Scientific knowledge Technical skill (manual dexterity) Communication (verbal, non-verbal and written) Decision-making Clinical reasoning	EPA 1: Briefly performs an anamnesis and focused physical examination of the airway
Identifies and describes signs and symptoms of airway impairment; recognizes needs and priorities in the development of actions relevant to forecasting devices, materials, and equipment needed to clear the airways; performs the manual airway opening techniques of head tilt-chin lift, jaw-thrust, and chin lift; performs the insertion techniques of temporary oropharyngeal cannula (Guedel) or nasopharyngeal cannula; and describes indications and contraindications for the use of these devices	Scientific knowledge Technical skill (manual dexterity) Communication (inter-professional, patient/family member) Leadership Decision-making Clinical reasoning	EPA 2: Performs manual opening and insertion of temporary airway maintenance devices
Recognizes the need and aspirates the airways with the type of material appropriate to the patient's clinical case (flexible or rigid), performs the pulse oximetry insertion techniques to check peripheral capillary oxygen saturation (SpO2), identifies factors (distal perfusion, low temperature, lesions, etc.) that can make it difficult to read peripheral capillary oxygen saturation (SpO2) in the oximeter, and applies the principles of biosafety in patient care with compromised airway	Scientific knowledge Technical skill (manual dexterity) Communication Leadership Decision-making Clinical reasoning	EPA 3: Recognizes the need for and the conduction of an intervention in airway aspiration

**Figure 2:** Association between competence frameworks to develop competences in the training of nurses to assist the airway of adult patients in urgency and emergency situations, essential competences and Entrustable Professional Activities (EPA 1, EPA 2 and EPA 3). Ribeirão Preto, SP, Brazil, 2017

Competence framework	Essential competences (Knowledge, Skill and Attitude)	Entrustable Professional Activities (EPAs)
Collaborates in or performs the insertion of supraglottic airway devices (laryngeal mask, laryngeal tube, double-lumen esophageal-tracheal tube) and performs thoracic auscultation to check device positioning	Scientific knowledge Technical skill (manual dexterity) Communication Leadership Team work Decision-making Clinical reasoning Safety	EPA 4: Collaborates or performs the insertion of supraglottic airway devices
Identifies factors related to airway impairment (signs of obstruction) or anatomical changes/abnormalities that suggest a difficult airway, performs airway intervention with signs of obstruction using manual opening techniques, insertion of temporary devices or aspiration of content, and recognizes the indications and contraindications and the techniques of advanced maneuvers to control airways such as nasotracheal and orotracheal intubation, cricothyroidostomy (puncture/surgical), tracheostomy.	Scientific knowledge Technical skill (manual dexterity) Communication Leadership Teamwork Decision-making Clinical reasoning Safety	EPA 5: Identifies, intervenes and indicates or contraindicates the techniques of advanced maneuvers to control the airway.
Collaborates with the team to perform advanced maneuvers to control the airways (preparation, testing and organization of the material, assistance with equipment preparation, patient positioning, preparation of medications, etc.)	Scientific knowledge Technical skill (manual dexterity) Inter-professional communication Decision-making Clinical reasoning Leadership	EPA 06: Collaborates with the team to perform advanced maneuvers to control the airway

## Discussion

The national emergency care system has presented innovative proposals and many organizational advances in relation to the definition of concepts and the incorporation of new technologies in health care services, aiming at improvements in the organization of network care^(^ 25 ^)^.

In this context, the need to systematize care for patients with airway involvement is essential to avoid aggravating situations that can be controlled, since the patients demonstrate a series of signs and symptoms that warn about the severity of their physiological health conditions and, for that moment, qualified professional intervention is fundamental for a positive care outcome^(^ 26 ^)^.

Airway management is fundamental to the care outcome; however, it is still very difficult for many health professionals to obtain sufficient experience in approaching the airways without adequate training^(^ 27 ^)^. This difficulty is a reality that affects the training of nurses because it practically happens based on their clinical experience in a relatively non-systematized learning process^(^ 5 ^)^.

On the other hand, it is known that the nurses who work in the care of these patients necessarily need specialized skills and knowledge, in order to present quick critical thinking in situations of imminent risk to life. High-level cognitive and emotional skills are associated with technical and relational dilemmas encountered daily in these settings^(^ 28 ^)^. Thus, the development and assessment of airway care management through an educational program with a structured curriculum is necessary, as this intervention is not yet standardized in the clinical practice among the professionals^(^ 5 ^)^.

However, much of the literature that addresses airway management in emergencies is associated with the medical field, and more specifically anesthetic^(^ 29 ^-^ 31 ^)^. Some studies have developed the theme with nurses; however, they limit this airway management to the interventions, such as artificial airway aspiration and care in the prevention of pneumonia associated with mechanical ventilation^(^ 32 ^-^ 33 ^)^.

Such data are significant, since Nursing makes up most of the contingent of health professionals and assumes day-to-day care for the patient, with managerial, assistance, educational and inter-professional responsibilities. However, what is commonly found are professionals working in the labor market who were trained urgently with the use of conservative teaching methodologies, in precarious laboratories, only limited to learning skills. In addition, during the training period of undergraduate Nursing students, as well as in other professions, some emergency interventions are rare in the clinical practice, which creates difficulties for the future professional in acquiring skills and incorporating critical-reflective thinking and decision-making in this area of performance^(^ 34 ^)^.

Systematic care for critically-ill patients is essential to avoid uncontrolled situations that can be stabilized. In order to provide high quality professional Nursing care, nurses must use a systematic and scientifically based approach, which includes assessing a patient's anatomical, physiological and neurological status. Physical examination, planning, implementation and evaluation of Nursing care require the development of nurses' knowledge, skills and attitudes^(^ 35 ^)^.

Allowing the training of nurses to provide adequate competence for the use of alternative devices for airway management, especially in emergency care, is an evident gain. The benefits that a supraglottic device can bring to the patient in an emergency situation during pre-hospital care can be numerous^(^ 36 ^-^ 37 ^)^, and the development of this skill during training is important, so much so that the researchers^(^ 38 ^)^ claim that endotracheal intubation is considered a "gold standard" for airway assistance. However, especially in pre-hospital care, it can become a complex psychomotor task for the professional in charge, causing complications in care, in addition to the fact that supraglottic devices, when used by non-competent professionals, also produce serious harms to the patients, which implies the relevance of the development and assessment of skills for this purpose^(^ 39 ^)^.

Emergency management of the airways must be performed by competent nurses, with knowledge, skills and attitudes, in which the nurse requires the development of professional characteristics such as quick decision-making, precise handling of the airways, leadership in crisis situations and managing their team during care^(^ 40 ^)^.

Among the factors that most contribute to incidents during airway management are faults in teamwork such as lack of professional roles, leadership deficiency, deficiencies in verbal and non-verbal communication between team members, in addition to lack of training and training assessment, lack of equipment and medications, and the patient's varied clinical conditions, among others^(^ 2 ^,^ 41 ^)^.

The study^(^ 40 ^)^ reports that nurses often face emergency situations, such as an unconscious patient with ventilatory difficulties. Faced with such situations, nurses must act immediately if an endotracheal intubation is necessary, demonstrating the competence on which medications, devices and equipment should be used during the intervention.

Competence-based training is able to improve the apprentice's performance, and its use in training and qualification can have an impact on the final results in the care of patients in acute or even critical situations, allowing for increased self-confidence and reduced insecurities^(^ 2 ^,^ 41 ^)^.

In this context, the definition of the competence frameworks allows the students to visualize their current development status and reflect on what the necessary behaviors are in their professional training^(^ 42 ^-^ 43 ^)^. In this way, the competence frameworks and EPAs can assist in curriculum development and assessment, helping to develop the qualities needed for a competent practice, although they are on a new path to education that has not yet fully developed, but is likely to affect the future of competence-based training in many countries^(^ 18 ^,^ 44 ^)^.

In this study, the EPAs were validated in simulated environments. It was noticed that many of the difficulties found by the evaluators in the use of the EPAs in simulated environments can be associated with the fact that the application of EPAs is a guide for the facilitators in the evaluation of the clinical practice. There were limitations regarding the number of experts participating in each phase of the study, difficulties in not finding studies on the theme on Nursing in national and international literature, nor current studies aimed at airway assistance in urgency situations and emergency and mainly in the training of nurses, which exalts the originality, but also the difficulty of obtaining other research studies that may confront the results found.

Therefore, new scientific studies are suggested to confirm the possibilities of the competence frameworks and EPAs and the real advantages for teaching and assessing competences for the training of nurses.

## Conclusion

The present study resulted in the construction of the competence frameworks and six EPAs, relating them to the domains of essential competences in the training of nurses to assist the airway of adult patients in urgency and emergency situations. The participation of experts in the construction and validation of this material was essential to guarantee the theoretical and practical relevance of the result.

Further studies are expected to carry out clinical validation during student practice to assess the effectiveness of the constructed and validated material. In addition, it is believed that the results of this study may allow the development of strategies for the development and assessment of student performance with impartial feedback.

## References

[1] Higginson R, Parry A, Williams M (2016). Airway management in the hospital environment. Br J Nurs.

[2] Cook TM, Woodall N, Frerk C (2011). Major complications of airway management in the UK: results of
the Fourth National Audit Project of the Royal College of Anaesthetists and
the Difficult Airway Society. Part 1: Anaesthesia. Br J Anaesth.

[3] Kennedy CC, Cannon EK, Warner DO, Cook DA (2014). Advanced Airway Management Simulation Training in Medical
Education: A Systematic Review and Meta-Analysis. Crit Care Med.

[4] Gruber E, Oberhammer R, Balkenhol K, Strapazzon G, Procter E, Brugger H (2014). Basic life support trained nurses ventilate more efficiently with
laryngeal mask supreme than with facemask or laryngeal tube
suction-disposable- a prospective, randomized clinical trial. Resuscitation.

[5] Kuszajewski ML, O'Donnell JM, Phrampus PE, Robey WC, Tuite PK (2016). Airway Management: A Structured Curriculum for Critical Care
Transport Providers. Air Med J.

[6] Sportsman S (2010). Competency education and validation in the United States: what
should nurses know?. Nurs Forum.

[7] Frenk J, Chen L, Bhutta ZA, Cohen J, Crisp N, Evans T (2010). Health professional for a new century: transforming education to
strengthen health systems in independent world. Lancet.

[8] Higginson R, Jones B, Davies K (2010). Airway management for nurses: emergency assessment and
care. Br J Nurs.

[9] Simpson T (2015). Airway management skills and knowledge for nurses. Br J Nurs.

[10] Baker PA, Riley RH (2011). Education in airway management. Anaesthesia.

[11] Bond W, Siegelman JN, Miller D, Cassara M, Barker L (2018). Simulation for Assessment of Milestones in Emergency Medicine
Residents. Acad Emerg Med.

[12] Ten Cate O, Chen HC, Hoff RG, Peters H, Bok H, Van Der Schaaf M (2015). Curriculum development for the workplace using Entrustable
Professional Activities (EPAs): AMEE Guide No. 99. Med Teach.

[13] Miranda FBG, Mazzo A, Pereira-Junior GA (2018). Construction and validation of competency frameworks for the
training of nurses in emergencies. Rev. Latino-Am. Enfermagem.

[14] Krupat E, Pelletier SR (2016). The development of medical student competence: tracking its
trajectory over time. Med Sci Educ.

[15] Wancata LM, Morgan H, Sandhu G, Santen S, Hughes DT (2016). Using the ACMGE milestones as a handover tool from medical school
to surgery residency. J Surg Educ.

[16] Lamba S, Wilson B, Natal B, Nagurka R, Anana M, Sule H (2016). A suggested emergency medicine boot camp curriculum for medical
students based on the mapping of Core Entrustable Professional Activities to
emergency medicine level 1 milestones. Adv Med Educ Pract.

[17] Breckwoldt J, Beckers SK, Breuer G, Marty A (2018). Entrustable professional activities: promising concept in
postgraduate medical education. Anaesthesist.

[18] Ten Cate O (2018). A primer on entrustable professional activities. Korean J Med Educ.

[19] Oliveira DC (2008). Theme/category-based content analysis: a proposal for
systematization. Rev Enferm UERJ.

[20] Fehring RJ (1987). Methods to validate nursing diagnoses. Heart Lung.

[21] Scarparo AF, Laus AM, Azevedo ALCS, Freitas MRI, Gabriel CS, Chaves LP (2012). Reflections on the use of Delphi technique in research in
nursing. Rev Rene.

[22] Oliveira AKA, Vasconcelos QLDAQ, Melo GSM, Melo MDM, Costa IKF, Torres GV (2015). Instrument validation for peripheral venous puncture with
over-the-needle catheter. Rev Rene.

[23] Association of American Medical Colleagues (2014). Core entrustable professional activities for entering residency:
curriculum developers' guide.

[24] Touchie C, Boucher A (2016). Entrustable professional activities for the transition from medical
school to residency.

[25] Morais LA, Martini JG, Lazzari DD, Vargas MAO, Backes VMS, Farias GM (2017). Urgency/Emergency course content in the education of generalist
nurses. Rev Min Enferm.

[26] Meyer G, Shatto B, Delicath T, Von Der Lancken S (2017). Effect of curriculum revision on graduates' transition to
practice nurse educator. Nurse Educ.

[27] Sun Y, Pan C, Li T, Gan TJ (2017). Airway management education: simulation based training versus
non-simulation based training- a systematic review and
meta-analyses. BMC Anesthesiol.

[28] Goldsworthy S (2016). Mechanical Ventilation Education and Transition of Critical Care
Nurses into Practice. Crit Care Nurs Clin North Am.

[29] Zoric L, Savoldelli GL (2015). Evidence base in airway management training. Trends Anaesth Crit Care.

[30] Awanee K (2010). Emergency airway management by non-anaesthetic
trainees. Resuscitation.

[31] Muratore S, Kim M, Olasky J, Campbell A, Acton R (2017). Basic airway skills acquisition using the American College of
Surgeons/Association for Surgical Education medical student simulation-based
surgical skills curriculum: Initial results. Am J Surg.

[32] Kjonegaard R, Fields W, King ML (2010). Current practice in airway management: A descriptive
evaluation. Am J Crit Care.

[33] Sole ML, Bennett M (2014). Comparison of airway management practices between registered
nurses and respiratory care practitioners. Am J Crit Care.

[34] Miranda FBG, Mazzo A, Pereira-Junior GA (2018). Use of high fidelity simulation in the preparation of nurses for
urgency and emergency care: scoping review. Sci Med.

[35] Tuzer H, Dinc L, Elcin M (2016). The effects of using high-fidelity simulators and standardized
patients on the thorax, lung, and cardiac examination skills of
undergraduate nursing students. Nurse Educ Today.

[36] Kurola J, Paakkonen H, Kettunen T, Laakso JP, Gorski J, Silfvast T (2011). Feasibility of written instructions in airway management training
of laryngeal tube. Scand J Trauma Resusc Emerg Med.

[37] Paal P, Herff H, Mitterlechner T, Von Goedecke A, Brugger H, Lindner KH (2010). Anaesthesia in prehospital emergencies and in the emergency
room. Resuscitation.

[38] Hasegawa K, Hiraide A, Chang Y, Brown DF (2013). Association of Prehospital Advanced Airway Management With
Neurologic Outcome and Survival in Patients With Out-of-Hospital Cardiac
Arrest. JAMA.

[39] Bernhard WB, Beres W, Timmermann A, Stepan R, Greim CA, Kaisers UX (2014). Prehospital airway management using the laryngeal tube: An
emergency department point of view. Anaesthesist.

[40] Han MJ, Lee JR, Shin YJ, Son JS, Choi EJ, Oh YH (2018). Effects of a simulated emergency airway management education
program on the self-efficacy and clinical performance of intensive care unit
nurses. Jpn J Nurs Sci.

[41] Thim T, Krarup NH, Grove EL, Rohde CV, Lofgren B (2012). Initial assessment and treatment with the airway, breathing,
circulation, disability, exposure (ABCDE) approach. Int J Gen Med.

[42] Ketterer AR, Salzman DH, Branzetti JB, Gisondi MA (2017). Supplemental milestones for emergency medicine residency
programs: a validation study. West J Emerg Med.

[43] Lomis KD, Russell RG, Davidson MA, Fleming AE, Pettepher CC, Cutrer WB (2017). Competency milestones for medical students: Design,
implementation, and analysis at one medical school. Med Teach.

[44] Touchie C, Ten Cate O (2016). The promise, perils, problems and progress of competency-based
medical education. Med Educ.

